# Tuberal Hypothalamic Neurons Secreting the Satiety Molecule Nesfatin-1 Are Critically Involved in Paradoxical (REM) Sleep Homeostasis

**DOI:** 10.1371/journal.pone.0052525

**Published:** 2012-12-27

**Authors:** Sonia Jego, Denise Salvert, Leslie Renouard, Masatomo Mori, Romain Goutagny, Pierre-Hervé Luppi, Patrice Fort

**Affiliations:** 1 Sleep-Waking Neuronal Networks, CNRS - UMR5292; INSERM - U1028, Lyon Neuroscience Research Center (CRNL), Lyon, France; 2 University Claude Bernard Lyon 1, Lyon, France; 3 University of Lyon, Lyon, France; 4 Department of Medicine and Molecular Science, Gunma University Graduate School of Medicine, Maebashi, Gunma, Japan; University of Cambridge, United Kingdom

## Abstract

The recently discovered Nesfatin-1 plays a role in appetite regulation as a satiety factor through hypothalamic leptin-independent mechanisms. Nesfatin-1 is co-expressed with Melanin-Concentrating Hormone (MCH) in neurons from the tuberal hypothalamic area (THA) which are recruited during sleep states, especially paradoxical sleep (PS). To help decipher the contribution of this contingent of THA neurons to sleep regulatory mechanisms, we thus investigated in rats whether the co-factor Nesfatin-1 is also endowed with sleep-modulating properties. Here, we found that the disruption of the brain Nesfatin-1 signaling achieved by icv administration of Nesfatin-1 antiserum or antisense against the nucleobindin2 (NUCB2) prohormone suppressed PS with little, if any alteration of slow wave sleep (SWS). Further, the infusion of Nesfatin-1 antiserum after a selective PS deprivation, designed for elevating PS needs, severely prevented the ensuing expected PS recovery. Strengthening these pharmacological data, we finally demonstrated by using c-Fos as an index of neuronal activation that the recruitment of Nesfatin-1-immunoreactive neurons within THA is positively correlated to PS but not to SWS amounts experienced by rats prior to sacrifice. In conclusion, this work supports a functional contribution of the Nesfatin-1 signaling, operated by THA neurons, to PS regulatory mechanisms. We propose that these neurons, likely releasing MCH as a synergistic factor, constitute an appropriate lever by which the hypothalamus may integrate endogenous signals to adapt the ultradian rhythm and maintenance of PS in a manner dictated by homeostatic needs. This could be done through the inhibition of downstream targets comprised primarily of the local hypothalamic wake-active orexin- and histamine-containing neurons.

## Introduction

It is accepted that the brainstem is sufficient for the generation of paradoxical (REM) sleep (PS). The leading hypothesis until the late 1990s indeed ascribed a pivotal role for PS onset to reciprocal inhibitory interactions between mesopontine cholinergic and monoaminergic neurons [1]. However, recent work led us to switch to an original GABAergic/glutamatergic hypothesis. Neurons within the pontine sublaterodorsal nucleus (SLD) that generate PS are glutamatergic. During waking and slow wave sleep (SWS), they are tonically inhibited by GABAergic inputs from the ventrolateral part of the periaqueductal gray and adjacent deep mesencephalic reticular nucleus. This inhibition must be removed to allow the SLD activation required for PS induction [2], [3], [4], [5], [6], [7]. For such a role, evidence has implicated the tuberal hypothalamic area (THA), upstream to this brainstem network, as an integrative center for PS regulation [5], [8]. Among the neurochemically-identified neurons within the THA, those secreting the melanin-concentrating hormone (MCH) have been recently highlighted in the sleep field since they express c-Fos protein during PS hypersomnia [9]. Electrophysiological recordings coupled with juxtacellular labeling confirmed that MCH-secreting neurons fire selectively during PS [10]. This observation, strengthened by the fact that intracerebroventricular (icv) administration of MCH enhances SWS and PS, points to a role of MCH signaling in sleep [9], [11], [12].

Of particular interest, Nesfatin-1, a recently discovered post-translational product of the nucleobindin-2 (NUCB2) gene, is strongly produced in hypothalamic areas including the THA, where it is notably co-expressed in all MCH-secreting neurons (MCH-Nesf-1+) [13], [14], [15]. This molecule secreted in brain and cerebrospinal fluid plays a role in appetite as a satiety molecule acting at picomole concentrations through hypothalamic leptin-independent mechanisms [16], [17], [18], [19], [20], [21]. Interestingly, this role in food intake is opposite to that of MCH [22], [23]. Recently published studies further associated Nesfatin-1 to other major physiological functions shared by MCH signaling such as mood regulation or fear-related responses. Indeed, both molecules are endowed with pharmacological properties known to increase anxiety [20], [22], [23], [24], [25], [26]. While the somnogenic potencies of MCH are documented [12], the ability of Nesfatin-1 to regulate sleep has not yet been investigated. To fill this gap in knowledge, we first assessed the impact on sleep of Nesfatin-1 signaling alterations in rats chronically prepared for icv pharmacological treatments combined with polysomnographic recordings. As a selective effect on PS was revealed, we then compared the c-Fos signal in Nesfatin-1-secreting neurons in experimental conditions designed to cause differential PS expression. The congruent pharmacological and functional data reported here provide the first evidence that the endogenous Nesfatin-1 tone strictly operated by MCH-Nesf-1+ neurons within the THA may be a key neurochemical component of the hypothalamic mechanisms regulating PS in a manner dictated by homeostatic needs.

## Materials and Methods

### Animals and Surgery

Animal experiments were carried out in strict accordance with the European Communities Council Directive (86/609/EEC) and the recommendations in the guide for the Care and Use of Laboratory Animals of the National Institutes of Health (NIH publication n° 85–23). Protocols were approved by the local committee on the Ethics of Animal Experiments of the University Claude Bernard, Lyon I (n° BH 2006–10).

Male Sprague-Dawley rats (Charles River, France), weighing 200–220 g (7–8 weeks old) when arriving in the lab facility, were placed under a controlled 12/12 h light–dark cycle (lights on at 7 am) at ambient temperature (22±1°C) for 10 days prior to surgery. Water and food pellets were available *ad libitum*.

All rats used in this work were chronically prepared under anesthesia (chloral hydrate, 400 mg/kg) for polysomnography; efforts were made to minimize suffering. Briefly, 3 cortical stainless steel screw electrodes and 2 neck muscle stainless wires were implanted to monitor electroencephalogram (EEG) and electromyogram (EMG) activity, respectively [3]. Rats planned for icv administration were also implanted with a guide cannula (Plastics one, VA, USA) placed above the right lateral ventricle (AP = −0.8 mm, ML = 1.7 mm, DV = 2.5 mm). Electrodes/wires soldered to a 6-pin connector and the guide-cannula were then fixed to the skull using Super-Bond (Sun Medical Co., Shiga, Japan) and acrylic cement. Following surgery, rats were allowed to recover and habituate to their environment for at least one week in single Plexiglas barrels before experiments began at 10–11 weeks age. During the recovery period rats were also handled daily for 30 minutes by the experimenter to acclimatize them to the experimental conditions. Once fully recovered and acclimatized to their environment, rats were continuously recorded until time of sacrifice.

### Drug Injections and Polysomnographic Recordings

Correct cannula positioning was first verified by an initial angiotensin II infusion (100 ng in 2 µl saline, Bachem, Switzerland) that induced a rapid dipsogenic response. The following acute pharmacological treatments were then conducted: 1) Synthetic peptide identical to rat Nesfatin-1 (50 and 250 pM, n = 12 and 10, respectively) *vs.* saline (5 µl, 1 µl/min) at 6 pm; 2) anti-Nesfatin Ab24 IgG (16 µg in 4.4 µl, n = 9; 8 µg in 2.2 µl, n = 7) *vs.* control rabbit IgG at 10 am; and 3) morpholino oligonucleotide (MON, 100 µg in 5 µl, Gene Tools, OR, USA) antisense against NUCB2 encoding gene (5′-ATGGTCCTCCACCTCA TCTTCAGAG-3′) *vs.* missense (5′-ATCGTGCTCCACGTCACTACACAG-3′) at 10 am. To avoid crossing effects, drug administrations were spaced apart by 3–4 days. Finally, 4 rats received the antisera treatment to block endogenous Nesfatin-1 signaling (control or Ab24 IgG) at the end of a selective deprivation of PS (3 pm) started at 9 am (see detailed protocol below). The dose of exogenous Nesfatin-1, Ab24 antiserum or MON antisense against NUCB2 was selected according to prior work completed using the same compounds [18]. According to this previous work, the chronic icv administration of MON antisense with mini-infusion pump at the dose of 40 µg/day is effective from the first day of treatment at inducing significant changes in food intake [18]. For the present acute treatment, we therefore choose to use a single larger dose of 100 µg of MON (x 2,5 *vs* 40 µg low dose previously tested) to rapidly disrupt the brain nesfatin-1 signaling. Exogenous Nesfatin-1, corresponding to residues 1–82 of the rat NUCB2, was synthesized by Yanaihara Institute Inc (Japan). The Ab24 antiserum was raised in rabbit against residues 24–38 of amino-acid sequences of rat NUCB2 and was thus also used for the immunodectection of Nesfatin-1 in brain sections (see below).

Immediately after treatment, rats were returned to their home barrel for recording. EEG and EMG signals were amplified, digitized at 200 Hz and collected continuously on a computer located in a room separate from the recording chamber *via* a CED interface using the Spike 2 software (Cambridge Electronic Design, UK). Three vigilance states (W, waking; SWS, Slow Wave Sleep; PS, paradoxical sleep) were visually identified and quantified *offline* in 5 sec epochs using custom-made scripts [3], [9].

During our experiments, we were not able to measure or estimate changes in the rat’s food intake and body weight likely induced by the pharmacological treatments with either Nesfatin-1, Ab24 or MON antisense, as previously demonstrated [18]. Indeed, our experimental conditions are only dedicated to chronic polysomnographic recordings with conventional Plexiglas barrels. Moreover, the variations of these metabolic parameters are in the range of hundreds of mg (less than 2 g) after acute pharmacological treatments, and only measurable by using metabolic cages. As this was not a focus of the present study, rats were fed *ad libidum* and undisturbed in their home barrel for 24 h after the treatment to avoid biased effects on vigilance state.

### PS Deprivation and Sleep Recovery

For this portion of the study, 27 rats were used. Our PS deprivation protocol was designed to obtain an elevation of PS amounts during the recovery period (also coined PS rebound) sufficient for inducing an expression of c-Fos in brain areas recruited during PS (see Results). Hence, we choose to deprive rats during the circadian period of highest PS pressure, from 9 am to 3 pm. Throughout the 6 h process the operator constantly scrutinized *online* EEG/EMG recordings on the computer screen, gently waking animals up (light noise or taping on barrels) whenever signs of PS were detected, i.e. muscle atonia concomitant to EEG activation with theta rhythmic activity for 5 sec. Attempts to enter in PS ( = awaking stimulations) were timely noticed and counted. At 3 pm, awakening stimulations were halted without perturbing the rats, and they were then allowed 2 h sleep recovery (n = 17, Recovery group). During the whole process (24 h baseline, 6 h deprivation and 2 h recovery), rats remained in their home barrels containing dry litter, with easy access to food/pellets and water. They were never handled until sacrifice. Two additional experimental groups were completed for the subsequent functional study (see below) including rats subjected to PS deprivation without opportunity for recovery sleep (n = 5, Deprivation group) or remaining undisturbed during the same period (n = 5, Control group).

### Functional Neuroanatomy with c-Fos Immunostaining

At 5 pm, rats designated for c-Fos immunostaining (n = 18 rats, see Results below) were anesthetized with a lethal dose of Nembutal (60 mg/kg) and perfused transcardially with a Ringer's lactate solution containing 0.1% heparin followed by 500 ml of an ice cold 4% paraformaldehyde fixative solution in 0.1 M phosphate buffer (PB; pH 7.4). After cryoprotection in PB containing 30% sucrose, brains were frozen and cut serially in coronal sections (25 µm thick). Free-floating sections were collected in PB containing 0.9% NaCl, 0.3% Triton X-100 (PBST) and 0,3% H_2_O_2_, rinsed two times in PBST and then stocked in PBST with 0.1% sodium azide (PBST-Az) at 4°C.

Sections were then submitted to the classical sequential incubations required for dual c-Fos/Nesfatin-1 immunostaining. They were first incubated in (i) a primary antiserum to c-Fos raised in rabbit (1/15000 in PBST-Az; Merck, USA) for 72 h at 4°C; (ii) a secondary anti-rabbit biotinylated IgG (1/1000 in PBST) for 90 min at room temperature; (iii) Avidin-Biotin-HRP complex (ABC, 1/1000; Elite Kit, Vector labs, CA, USA) in PBST for 90 min at room temperature; and (iv) 0.05 M Tris-HCl buffer (pH 7.6) containing 0.025% 3,3′-diaminobenzidine-4 HCl (DAB; Sigma, France), 0.003% H_2_O_2_ and 0.6% nickel ammonium sulfate for 20 min. The staining appeared as a dense black nuclear coloration of c-Fos-immunoreactive (Fos+) neurons. The antiserum to c-Fos was made against a synthetic peptide corresponding to the N-terminal part (residues 4–17) of human Fos. This part of the protein displays 100% homology between human and rat and no homology with Fos-related antigens such as Fos B, Jun B, Fra-1 and Fra-2 (Blast 2 sequences, NCBI).

After extensive rinses in PBST-Az (2–3 days at 4°C), pretreated sections were submitted to sequential incubations for Nesfatin-1 immunodetection with the Ab24 rabbit primary antiserum (1/30000 in PBST-Az for 72 h at 4°C) [15]. Using Tris-HCl solution without Nickel, the orange-brown colored reaction product appeared in cytoplasm and primary dendrites of Nesfatin-1 immunopositive (Nesf-1+) neurons. The Ab24 antibody was raised against a peptide corresponding to NUCB2 24–38 aa residues and thus recognizes the whole NUCB2/Nesfatin [18]. It demonstrated a high specificity and selectivity for native Nesfatin-1, without cross-reactivity with co-expressed neuropeptides in the paraventricular, arcuate and supraoptic nuclei (leptin, alpha-MSH or NPY) or the tuberal hypothalamic area (as CART, MCH, NEI and NGE) [15].

### Cell Counts and Analysis

A qualitative microscopic observation of immunostained sections from Forebrain to Brainstem levels clearly revealed that double-labeled (Fos+/Nesf-1+) cells were exclusively observed within the THA, independent of the experimental condition considered (Control, Deprivation and Recovery). Indeed, Fos+/Nesf-1+ neurons were never found in other brain areas known to contain large amounts of Nesf-1+ neurons including the paraventricular, arcuate or supraoptic nuclei within the hypothalamus, the locus coeruleus or the nucleus of the solitary tract within the brainstem [13], [14], [15], [18]. Hence, our quantitative analyses were focused on the THA for the 5 rats of the Control group, 5 rats of the Deprivation group and for 8 rats (of 17) from the Recovery group, selected according to the PS rebound they experienced during recovery (see Results). For each rat of this sample (n = 18 rats), double-labeled sections taken every 300 µm throughout the THA extent (n = 8 sections) were analyzed with an Axioskop 2 plus microscope (Zeiss, Germany) equipped with a motorized X–Y-sensitive stage and a video camera connected to a computerized image analysis system. The plotting of Fos+, Nesf-1+ and Fos+/Nesf-1+ neurons was done for one brain side using Mercator v.2 software (ExploraNova, France). After plotting, the same sections were counterstained with neutral red to draw boundaries of each discrete nucleus forming THA for precise cell mapping and counting. When a structure was present over several sections, neurons counted across the sections were summed to obtain the relative cell amounts encountered for each unilateral nucleus *per* rat. Mean cell amounts and percentages of Fos+/Nesf-1+ *vs.* singly Fos+ and Nesf-1+ stained cells (± SEM) were methodically calculated for each THA nucleus and experimental condition. According to the atlases of the rat hypothalamus [27], [28], labeled cells were plotted in the anterior hypothalamic nucleus (AH), the dorsal hypothalamic area (DA), the dorsomedial hypothalamic nucleus (DMH), the zona incerta (ZI), the subincertal nucleus (Subl), the lateral hypothalamic area (LH), the perifornical hypothalamic nucleus (PeF), the posterior hypothalamic area (PH) and the tuber cinereum (TC). However, to minimize cell estimation errors and to simplify the report of anatomical data, we completed our quantitative and correlative analyses by considering the THA and its lateral half (L-THA) as unique anatomical structures. The latter comprises more than 70% of the whole Nesfatin-1-expressing neurons of the THA [15]. The L-THA includes the ZI, Subl, LH and PeF nuclei.

Photomicrographs were taken with a CCD Color 10-bit QiCam camera. They were imported into Adobe Photoshop 7.0, digitally adjusted for brightness and contrast, and were assembled into plates at a resolution of 300 pi.

### Statistical Analysis

Amounts (in min) of W, SWS and PS for each pharmacological treatment were expressed as mean ± SEM. To detect statistical significance, data were analyzed as follows: for each vigilance state (W, SWS, PS) the effect of treatment (saline *vs*. Nesfatin-1, non-immune *vs.* Nesfatin-1 antisera or missense *vs.* antisense MON) was tested using a two-way analysis of variance (ANOVA) followed by a *post hoc* Bonferroni test to identify significant pairwise differences if appropriate. The cumulative amount of vigilance state at a specific time was tested with one-way ANOVA or paired t-test. Each animal served as its own control. Characterization of the PS deprivation and the ensuing recovery was done using a two-tailed paired t-test. Significance was set at p<0.05. The correlation between the number of Fos+/Nesf-1+ neurons and PS quantities was done using linear regression analysis. All statistical analyses were performed using GraphPad Prism 4.0.

## Results

### Endogenous Nesfatin-1 Signaling Modulates PS

**Figure 1:**
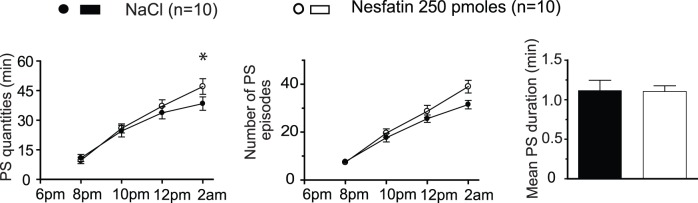
Exogenous Nesfatin-1 slightly up-regulates PS. Effects on PS of acute icv administration of a high dose of Nesfatin-1 (*vs.* saline, n = 10). Note the modest effect on cumulative PS amounts. The quantitative analysis is organized into 2 h blocks during the first 8 h following administration of Nesfatin-1 (open circles) or saline (filled circles). Cumulative PS quantities (left panel), number of PS bouts (second panel) and a histogram comparing the duration of PS bouts calculated over the 8 h time period post-treatment (*vs.* control) (right panel) are shown. Values are presented as mean ± SEM and significance values indicated for individual points *vs.* control with * p<0.05; ** p<0.01, *** p<0.001. For the first two columns, a two factor (time×treatment) within subject ANOVA followed by *post hoc* Bonferonni test was utilized, while in the third column statistics were performed using a two tailed paired t-test.

We first examined how vigilance states were affected following the administration of exogenous Nesfatin-1 (50 or 250 pM) 1 h before dark onset, when SWS and PS amounts naturally decrease (active circadian period). For both doses tested, no effect of Nesfatin-1 treatment was noticed on W ([F_(2,132)_ = 0.1472; p = 0.8633]), SWS ([F_(2,132)_ = 0.2415; p = 0.7858]) or PS amounts [F_(2,132)_ = 1.168; p = 0.3141]) compared to saline injected control conditions. However, significant increases in both the cumulative PS quantities (+22,5% *vs.* saline) and the mean number of PS bouts (+25,8% *vs.* saline) were observed over the 8 h post-treatment period for the higher dose (Figure 1). No effect on SWS amounts was noticed over the same period (173.5±9.7 min for Nesfatin-1 *vs.* 173.5±11.6 min for saline).

**Figure 2:**
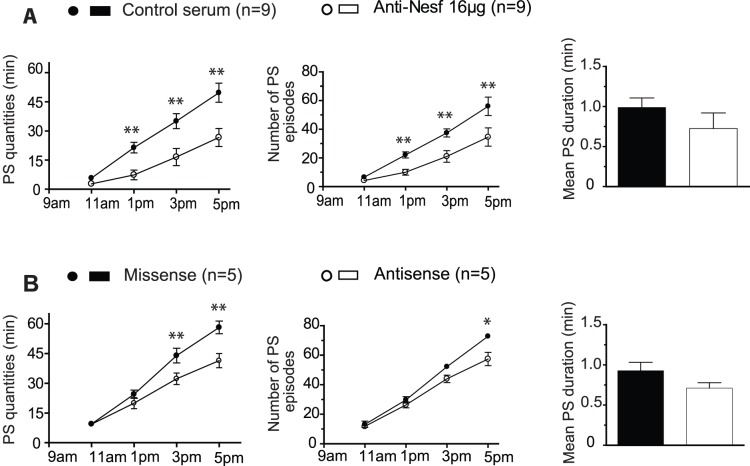
Disrupting endogenous Nesfatin-1 signaling suppressed PS. Effects on PS of acute icv administration of Nesfatin-1 antiserum (*vs.* normal serum, n = 9, **A**) or NUCB2 antisense (*vs.* missense, n = 5, **B**). The pharmacological disruption of endogenous Nesfatin-1 signaling with both compounds induced a significant and selective PS suppression. For each drug tested the quantitative analysis is organized into 2 h blocks during the first 8 h following administration of Nesfatin-1 (open circles) or saline (filled circles). Cumulative PS quantities (first column), number of PS bouts (second column) and a histogram comparing the duration of PS bouts calculated during the period of maximal drug effect (*vs.* control) for the first 6 h for antiserum and over 4 h from 2 to 6 h post-treatment for antisense (third column) are shown. Values are presented as mean ± SEM and significance values indicated for individual points *vs.* control with * p<0.05; ** p<0.01, *** p<0.001. For the first two columns, a two factor (time×treatment) within subject ANOVA following by *post hoc* Bonferonni test was utilized, while in the third column statistics were performed using a two tailed paired t-test.

To further investigate the PS-enhancing potency of Nesfatin-1, we assessed how sleep was affected by the acute blocking of endogenous signaling through the infusion of a specific antiserum [18]. Experiments were completed 1 h after light onset, when SWS and PS amounts naturally increase (rest circadian period). In this experimental condition, a significant dose-dependent effect of the treatment was found for PS ([F_(2,88)_ = 11.09; P<0.001]) and W ([F_(2,88)_ = 3.170; P = 0.0468]) but not for SWS ([F_(2,88)_ = 1.931; P = 0.1511]). For the higher antiserum concentration (16 µg), a *Post hoc* analysis indeed revealed that the antiserum disturbed PS during the first 4 h (Figure 2A). This effect resulted in a significant reduction of both cumulative PS amounts (−46,4% *vs.* normal serum) and mean number of PS episodes during the 8 h post-treatment period (−38,3% *vs.* normal serum). The mean duration of PS bouts over the first 6 h post-treatment also tended to be shortened compared to control (Figure 2A). No impact on cumulative SWS quantities was noticed over the same period (283.0±13.9 min for Nesfatin-1 antiserum *vs.* 281.1±9.6 min for normal serum).

An alternative strategy to acutely decrease endogenous Nesfatin-1 tone consisted of icv infusion of a single high dose of NUCB2 antisense (AS, 100 µg) 1 h after light onset. Treatment with NUCB2 antisense at this dose was previously shown to rapidly prevent NUCB2/Nesfatin translation in the brain of freely moving rodents [18]. As observed for the antiserum treatment, NUCB2 antisense administration resulted in a significant decrease in PS quantities ([F_(1,24)_ = 14.72; P = 0.0008]) without significant changes in SWS ([F_(1,24)_ = 0.006143; P = 0.9395] or W quantities [F_(1,24)_ = 0.4971; P = 048768]). *Post hoc* analysis showed that the effect occurred 6 hours after the AS administration compared to missense (MS) treatment (Figure 2B). This suppressive effect on PS (−27,4% *vs.* MS over 8 h) is mainly ascribable to a significant reduction in the number of PS episodes (−21,2% *vs.* MS). A shorter mean duration of PS bouts was also noticed during the effect, although this observation was not significant (Figure 2B). In the other condition tested, AS treatment did not disturb SWS amounts (228.5±8.8 min for AS *vs.* 233.2±10.3 min for MS).

These consistent pharmacological data support the contribution of Nesfatin-1 signaling to the promotion of PS without any effect on SWS. This implies that native Nesfatin-1 is locally released by neurons that must increase their activity selectively during PS prior to downstream somnogenic action.

### 6 h of PS Deprivation is Sufficient for Inducing a Robust PS Rebound

A way to identify these PS-activated neurons supplying Nesfatin-1 is to compare the expression of c-Fos used as a functional marker in Nesf-1-immunoreactive cell groups in rats that experienced different quantities of PS before sacrifice. This marker has been widely used by many groups to visualize neuronal activation across the sleep-waking cycle [3], [4], [6], [9], [29], [30], [31], [32], [33], [34], [35], [36], [37], [38], [39], [40], [41]. Hence, we designed a short lasting (6 h) protocol for selective PS deprivation and assessed its efficiency for inducing an increase of PS amounts (also coined PS rebound) during recovery (2 h).

**Figure 3:**
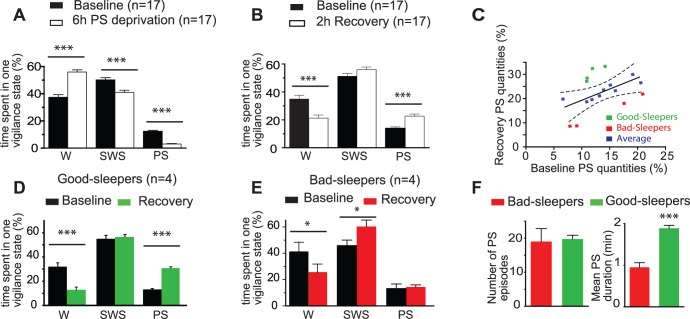
Six hours of PS deprivation is sufficient to achieve a robust PS rebound. Mean (± SEM) percentage of time spent in Waking (W), Slow Wave Sleep (SWS) and PS by the same group of rats (n = 17) during selective PS deprivation for 6 h (*vs.* Baseline, from 9 am to 3 pm; **A**) and the following 2 h recovery period (*vs.* Baseline, from 3 to 5 pm; **B**). The percentage of time spend in PS during deprivation is significantly reduced compared to baseline, at the expense of W (**A**, *** p<0.001). During the recovery period, the percentage of time spent in PS is almost doubled compared to baseline, to the detriment of W while SWS remained unchanged (**B**, *** p<0.001). **C**- Diagram illustrating the global linear relationship between the percentage of time spent in PS by each rat used (n = 17) during the 2 h recovery period following PS deprivation (in ordinates) and the corresponding baseline period (in abscises, 3–5 pm). With a 95% confidence interval, two groups of rats could be segregated: the so-called Good-Sleepers (n = 4; green squares) and Bad-Sleepers (n = 4; red squares). **D** and **E**, histograms comparing the percentage of time spend in W, SWS and PS during 2 h recovery period (*vs.* baseline, 3–5 pm) by the Good- (**D**, green) and Bad-Sleepers (**E**, red). Although submitted to the same deprivation protocol, Good-Sleeper rats experienced an intense PS rebound (x 2,5 *vs.* baseline PS values, **D**) while Bad-Sleepers did not (no significant difference with baseline PS values, **E**). As shown by histograms in **F**, the lack of PS rebound in Bad-Sleepers is only due to a shorter mean duration of PS bouts while the mean number of PS episodes was similar for both rat groups. Data are presented as mean ± SEM and significance values indicated for individual points *vs.* control with * p<0.05; ** p<0.01, *** p<0.001 using two tailed paired t-test.

As illustrated in the Figure 3A, the proportion of time spent in PS was dramatically reduced in the deprived rats compared to baseline (n = 17, p<0.0001). Only increases in short-lasting (< 5 sec) attempts to enter in PS were detected over the 6 h deprivation course, an index for the growing homeostatic pressure for PS (PS bouts in baseline *vs.* PS attempts during deprivation: 35.8±2.0 *vs.* 58.9±7.3, n = 17, p<0.0001). The percentage of SWS was also significantly reduced at the expense of the W fraction (−18,4% and +49,8% of the SWS and W amounts compared to baseline values, respectively). During the 2 h recovery following PS deprivation (Figure 3B), we observed a significant increase of the PS percentage, equivalent to an increase of +63,4% of PS amounts experienced by rats (n = 17) compared to their baseline values. The W amounts were significantly reduced (−39% *vs.* baseline, p<0.0001), while the quantities of SWS were similar to baseline ones. As a result, a short PS-deprivation of only 6 h is sufficient for inducing a PS rebound for 2 h.

An in-depth analysis of polysomnographic data from the individual rats led us to observe that PS quantities during recovery correlated positively with baseline PS amounts. As shown in Figure 3C, there was however some heterogeneity in behavioral responses to the PS deprivation. Based on this observation we discriminated two types of outliers using a 95% confidence interval from the former rat sample (n = 17): so-called good- and bad-sleepers (GS and BS respectively, n = 4 for each) [42], [43], [44]. Indeed, when comparing sleep parameters during the 2 h of recovery, the two discriminated GS and BS groups clearly behave in a different way. GS rats showed a percentage increase in time spent in PS, equivalent to an increase of +133,6% of PS amounts compared to baseline values (Figure 3D; [F_(1,6)_ = 149.003, p<0.0001]). This was concomitant to a significant decrease in W proportion [−60,2% of W amounts; F_(1,6)_ = 19.788, p = 0.0043]. Remarkably, SWS was not modified [F_(1,6)_ = 0.170, p = 0.6942]. As shown in Figure 3E, BS rats, on their own, did not recover efficiently from deprivation since the percentage of time spent in PS only faintly increased compared to baseline ([F_(1,6)_ = 0.287, p = 0.6113]; +8,5% of PS amounts *vs.* baseline). This lack of PS rebound was balanced by an increase of SWS proportion (F_(1,6)_ = 0.601, p = 0.4667; +32,2% of SWS amounts *vs.* baseline) and a decrease of W amounts (F_(1,6)_ = 0.741, p = 0.4677; −38,1% of W amounts *vs.* baseline). The more pronounced PS rebound in GS rats was due to a significantly longer duration of the PS bouts than those experienced by BS rats over the 2 h recovery, while the mean number of PS episodes similarly increased for both groups of rats compared to their respective baseline values (Figure 3F).

Of particular importance, the cumulative number of awaking stimulations applied by the operator over the PS deprivation was not significantly different between GS and BS rats (data not shown), indicating that both groups behave similarly during the experimental process with no evident difference in required deprivation intervention.

### The Recruitment of Nesfatin-1-expressing Neurons from the THA is Positively Correlated to PS Amounts

We next compared the expression of c-Fos, used as a marker of neuronal activation in Nesf-1-immunoreactive neurons in the GS and BS rats subjected to the same privation/recovery protocol (n = 4 for each), and in two additional rat groups, i.e. control and 6 h PS-deprived rats (n = 5 for each). As reported in Table 1, these 4 experimental groups greatly differed in the percentage of time spent in PS during the 2 h prior to sacrifice while SWS amounts were remarkably homogenous. This approach gave us a valuable opportunity to correlate the actual PS and SWS quantities with the recruitment level of brain Nesfatin-1 neurons. Indeed, the peak of c-Fos expression in response to sleep related changes, though varying depending on neuronal phenotype, has been estimated to be 30–70 min [39], [45], [46], a timeframe that perfectly matches the present experiments.

**Table 1:** Correlation between sleep quantities (SWS and PS) and consequent c-Fos expression in Nesfatin-1-immunoreactive cell bodies within THA for the 4 experimental groups considered, i.e. Control, PS deprived, Bad-Sleeper and Good-Sleeper rats.

		Control	PS deprivation	PS recovery	
				Bad-Sleepers	Good-Sleepers
**Sleep**	**% PS**	13.5±0.9	4.9±1.2**	14.3±1.4^a^	30.9±1.1***^a,c^
	**% SWS**	50.5±0.2	55±4.1	60.3±4.4	56.5±2.3
**THA**	**Fos+**	225,7±28,6	635±40,7***	585,3±74,7**	737,5±84,6***
	**Fos/Nesf-1+**	11,2±2,9	4,4±2,6	48,3±27	194,5±22,3***^a,c^
**ZI-Subl**	**Fos+**	55,2±8,8	110,2±14,5	147,5±23,2*	176,3±24,2**
	**Fos/Nesf-1+**	3,6±0,6	0,4±0,4	14±7	61,3±12,5***^a,c^
**PeF-LH**	**Fos+**	80,7±13,0	216,6±12,7***	199±24,9**	261,3±27,5***
	**Fos/Nesf-1+**	5,6±1,5	1,8±1,2	22,8±12,9	101,25±11,1***^a,c^
**L-THA**	**Fos+**	135.9±19.9	326.8±24.7**	346.5±43.8**	437.5±48.8***
	**Fos/Nesf-1+**	9.2±2.1	2.2±1.6	36.8±19,9	162.5±19.1***^a,c^

For each experimental condition, the percentage of time spent in SWS and PS scored per 5-sec epoch during the last 2 h before sacrifice is reported (mean ± SEM, n = 5 rats for Control and PS deprivation, n = 4 rats for both PS recovery samples). Then, the number (mean ± SEM) of Fos+, Nesf-1+ and Fos+/Nesf-1+ double-labeled neurons was estimated, bilaterally, for each discrete THA nucleus known to contain Nesf-1+ neurons (see Methods for detailed quantification). Here are reported quantitative data for single-labeled Fos+ and double-labeled Fos+/Nesf-1+ cell bodies when considering either the whole THA and separately, the lateral half of THA (formed by the ZI-Subl and PeF-LHA areas) which contains the majority of the THA Nesf-1+ neurons. Significance values are:

* p<0.05, **p<0.01, ***p<0.001 *vs.* control; ^a^ p<0.001 *vs.* privation and ^b^ p<0.05, ^c^ p<0.001 between bad sleepers and good sleepers, two-way ANOVA followed by a Bonferonni *post hoc* test.

**Figure 4:**
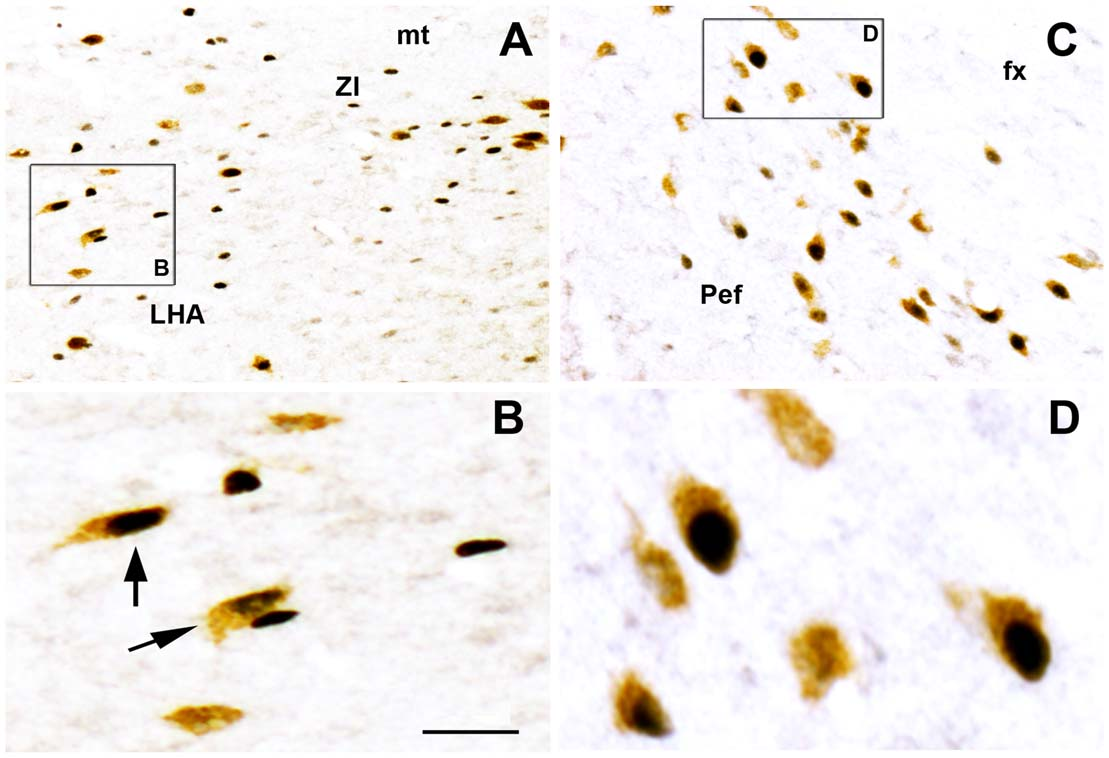
Nesfatin-1 immunoreactive neurons strongly express c-Fos during PS rebound. Photomicrographs of a double-immunostained frontal section at the THA level from a representative Good-Sleeper rat illustrating the high number of Fos+/Nesf-1+ double-labeled cells (arrows) within ZI (**A**, enlargement in **B**) and PeF (**C**, enlargement in **D**). Double-labeled neurons are easily recognized by their cytoplasm diffusely colored in brown (for Nesfatin-1 with DAB histochemical revelation) and their nucleus densely labeled in black (for c-Fos with Ni intensified DAB histochemical revelation). *Bars:* **A** and **C**, 200 µm; **B** and **D**, 20 µm. *Abbreviations*: fx, fornix; LHA, lateral hypothalamic; mt, mamillo-thalamic tract; Pef, perifornical nucleus; Subl, subincertal nucleus; ZI, zona incerta.

**Figure 5:**
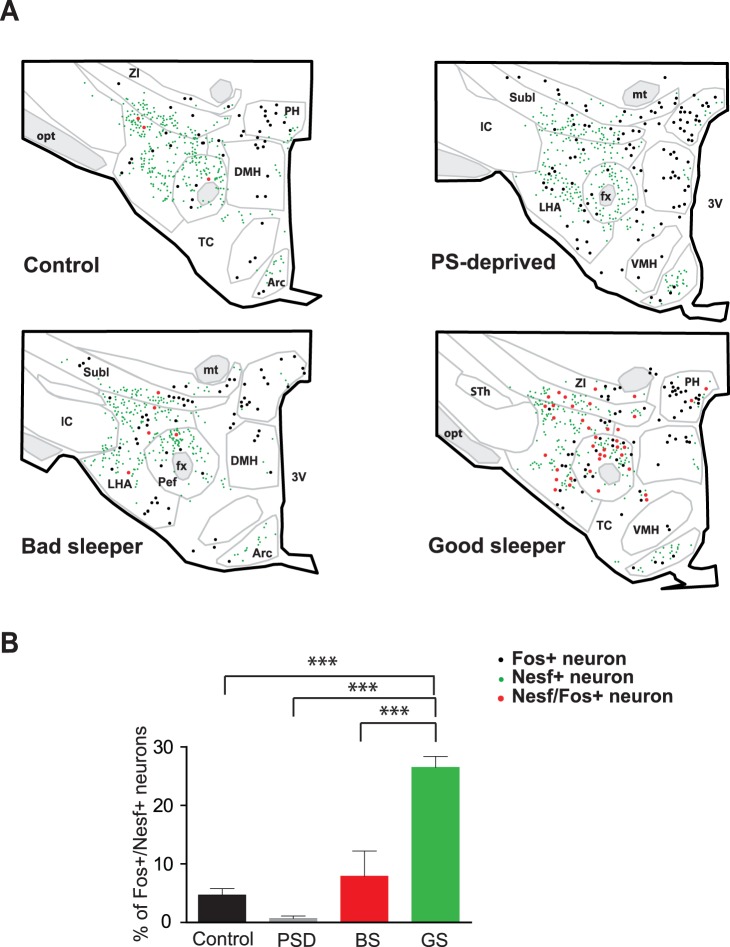
Nesfatin-1 neurons within THA are recruited during PS. A - Schematic drawings of frontal sections at the same THA level (AP = −3,30 mm according to Paxinos and Watson’s atlas) comparing the distribution of the Nesf-1+ (green dots), Fos+ (black dots) and Fos+/Nesf-1+ double-labeled (red dots) cell bodies plotted in a representative rat issued from the Control, PS-deprived, Bad-Sleeper or Good-Sleeper experimental samples. Each dot represents one labeled cell body. Notice the higher number of Fos+/Nesf-1+ neurons (red dots) in the ZI-Subl and the PeF-LHA areas in Good- compared to Bad-Sleepers. Both experimental groups were submitted to the same PS deprivation protocol and significantly differed only in PS amounts experienced during the last 2 h before sacrifice. **B-** Histogram showing that the mean percentage (± SEM) of Fos+/Nesf-1+ (*vs.* single Fos+) neurons is significantly higher in rats with intense PS rebound (Good-Sleepers, GS, n = 4) compared to the other three experimental conditions (Control, n = 5; PS-deprived, n = 5; Bad-Sleepers, n = 4). Data are presented as mean ± SEM. *** p<0.001 using a one way factor between subject ANOVA and *post hoc* Bonferonni test. *Abbreviations*: 3V, third ventricle; Arc, arcuate nucleus; DMH, dorsomedial hypothalamic nucleus; fx, fornix; IC, internal capsule; LHA, lateral hypothalamic; mt, mamillo-thalamic tract; opt, optic tract; Pef, perifornical nucleus; PH, posterior hypothalamic nucleus; Subl, subincertal nucleus; TC, Tuber cinereum; VMH, ventromedial hypothalamic nucleus; ZI, zona incerta.

Regardless of the experimental condition considered, Nesfatin-1 neurons positive for c-Fos (Fos+/Nesf-1+) were found exclusively within the tuberal hypothalamus area (THA) (Figures 4 and 5). Such double-labeled neurons were never encountered in other hypothalamic (i.e. paraventricular, supraoptic or arcuate nuclei) or brainstem nuclei known to contain large contingents of Nesf-1-immunoreactive neurons **(**data not shown**)**. For each discrete THA nucleus previously described as containing Nesfatin-1 neurons, and for each of the 4 experimental conditions, we consistently quantified the number and percentage of Fos+, Nesf-1+ and Fos+/Nesf-1+ neurons. However, to shorten the description of results, we only report in Table 1 pooled counting by considering the whole THA and its lateral parts (L-THA), the latter containing more than 70% of the THA Nesf-1+ neurons.

Within both the THA and L-THA, we encountered a significantly higher number of Fos+/Nesf-1+ neurons in GS rats than in BS and control rats, while rare double-labeled cells were seen in deprived rats (Table 1, Figures 4 and 5A). In GS rats, ∼27% of Fos+ neurons were double-labeled for Nesf-1, a proportion significantly higher to that observed for BS rats (∼8%) or control rats (∼5%) (Figure 5B). Within the L-THA, these proportions in the perifornical and lateral hypothalamic nuclei (PeF-LH) reached ∼39% of the Fos+ neurons in GS compared to ∼11% in BS, ∼6% in control and less than 1% in PS-deprived rats.

When considering each rat from the present 4 experimental groups individually (n = 18 rats), we further found that the number of Fos+/Nesf-1+ neurons in THA or L-THA displayed a positive correlation with the amounts of PS achieved during the last 2 h prior to sacrifice (THA: r^2^ = 0.6951, p = 0.0107; L-THA: r^2^ = 0.6934, p = 0.0103). In contrast, this number of Fos+/Nesf-1+ neurons was not correlated to SWS (THA: r^2^ = 0.007, p = 0.8429; L-THA: r^2^ = 0.008, p = 0.8386) or W amounts (THA: r^2^ = 0.3841p = 0.102, L-THA: r^2^ = 0.3877, p = 0.0992). Similar positive correlations between PS amounts and the percentage of Nesfatin-1 neurons labeled for c-Fos were obtained for the THA, L-THA or each discrete THA area taken individually (data not shown). This suggests a relatively homogeneous activation of the Nesf-1+ neurons associated with PS quantities, regardless of their localization within the THA region. Indeed, ∼22% of Nesfatin-1 neurons in each THA area were Fos-labeled in GS rats, ∼5% in BS, ∼2% in control and less than 0,5% in deprived rats.

Taken together, the present functional data demonstrate that Nesfatin-1 neurons selectively activated during PS are only found within the THA. Furthermore, the recruitment of these hypothalamic neurons is correlated to PS quantities but independent of either SWS or W amounts. Hence, these THA neurons could be the source of endogenous Nesfatin-1 involved in PS regulation.

### Nesfatin-1 Signaling is Involved in Mechanisms of PS Rebound

**Figure 6:**
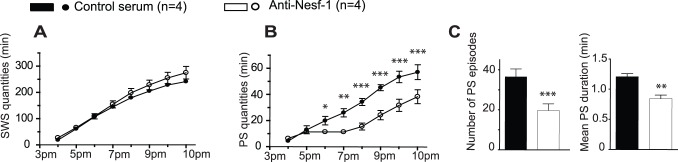
Nesfatin-1 signaling is involved in mechanisms of PS homeostasis. Graphs illustrating the cumulative amounts of SWS (**A**) and PS (**B**) over the first 8 h following an acute icv infusion of Nesfatin-1 antiserum (*vs.* normal serum in the same rats) done immediately following a 6 h period of PS deprivation (3 pm). Note the PS suppression during a time period of 2 h (5–7 pm) and thus the lack of the expected PS rebound after such deprivation (**B**, *vs.* normal serum treatment). Kinetics of SWS amounts during recovery remains unaffected (**A**). As shown in graph **C**, this suppressive effect is due to a decrease of both the mean cumulative number and the mean duration of the PS bouts, calculated over the first 6 h following treatment compared to control recovery in the same rats. Data are presented as mean ± SEM and significance values indicated for individual points *vs.* control with * p<0.05; ** p<0.01, *** p<0.001 using a 2 way-factor (time×treatment) within subject ANOVA and *post hoc* Bonferonni test. For C, two tailed paired t-test.

Endogenous Nesfatin-1 controls PS amounts. Further, Nesfatin-1 neurons from the THA are specifically activated during PS and their recruitment is correlated to PS amounts. This suggests that Nesfatin-1 signaling might be engaged in physiological situations of elevated PS needs, such as during PS rebound. To assess this possibility, we examined whether an injection of Nesfatin-1 antiserum at the end of a 6 h-PS deprivation prevents the expected PS rebound (Figure 6). An effect of the Nesfatin-1 antiserum treatment (16 µg *vs.* control antiserum, n = 4) was observed for both SWS and PS during sleep recovery ([F_(1,36)_ = 4.135; p = 0.0494] and [F_(1,36)_ = 14.04; p = 0.0006] respectively) but not for W ([F_(1,36)_ = 0.03499; p = 0.8527]). However, *post hoc* analysis revealed that cumulative PS, but not SWS amounts, were significantly affected over the first 4 h compared to the same period after normal serum treatment (Figure 6A; −56,2% *vs.* control recovery). An analysis hour *per* hour further revealed a largely complete PS withdrawal during 2 h (5–7 pm) after Nesfatin-1 antiserum treatment. Interestingly, the cumulative PS amounts did not reach normal values indicating that the PS debt induced by deprivation was still not compensated, even 8 hours after antiserum treatment (Figure 6B). As shown in Figure 6C, the debt of PS during recovery is due to a significant reduction of both PS bout number and their mean bout duration (*vs.* control recovery). Taken together, these results indicate that Nesfatin-1 facilitates the homeostatic response to PS deprivation.

## Discussion

This study is the first to show that Nesfatin-1 is endowed with somnogenic properties. By combining pharmacology and functional neuronanatomy, we indeed demonstrated that endogenous Nesfatin-1 signaling is involved in PS regulation, a role owed to Nesfatin-1 secreting neurons restricted to the tuberal hypothalamus (THA).

The strongest pharmacological evidence was achieved with experiments designed to acutely block Nesfatin-1 function by interrupting the endogenous signaling using two potent tools with different modes of action [18]. With either antiserum against Nesfatin-1 or antisense against NUCB2 encoding gene, a similar down-regulation of PS was indeed observed, although the latency and kinetics of the observed effects slightly differed between both compounds, likely due to their different mode of action. Furthermore, the antiserum administration performed after PS deprivation designed to elevate homeostatic PS needs attenuated the expected ensuing PS rebound. These effects on PS consistently reflected a decrease in the number of episodes concomitant to their shortened duration. Additionally, exogenous Nesfatin-1 induced a significant but faint PS up-regulation at high concentration only. One may suggest a rapid degradation of the neuropeptide before reaching downstream targets involved in PS that could be distant from the lateral ventricles. A more likely explanation is that the remarkably high level of PS amounts scored after control injection might have masked a stronger effect. This potential ceiling effect could also explain the inability to generate PS above physiological needs at the onset of the dark phase, when rats usually awaken and become active. It should be further kept in mind that SWS was not affected by any of the pharmacological treatments, and that the induced modifications on PS were counterbalanced only by fine adjustments of W amounts. This slightly differs from the somnogenic effects of MCH, previously reported to induce not only a PS hypersomnia but also a slight increase of SWS amounts when infused icv in rats [9]. In line with these results, the disruption of MCH transmission by subcutaneous treatment with MCH-R1 antagonists decreased dose-dependently deep SWS and PS, a change that was compensated by an increase of W in rats [47]. Taken together, these results suggest that MCH may promote total sleep while Nesfatin-1 signaling may be specifically engaged during PS, with a role in both its ultradian rhythm and homeostatic regulation.

This implies that the brain concentration of circulating Nesfatin-1 should increase during PS compared to SWS, proportional to PS amounts. This hypothesis was supported by the pattern of c-Fos expression in Nesf-1+ neurons relative to PS amounts prior to sacrifice. First, we showed that regardless of the experimental conditions, the THA is the unique brain area containing Nesf-1+ neurons positive for c-Fos. We further demonstrated that the recruitment of THA Nesf-1+ neurons is positively correlated to PS and not correlated to SWS amounts, meaning that as rats spent more time in PS the proportion of the Nesf-1+ neurons activated within the THA became more pronounced. Hence, Nesf-1+ neurons were not recruited during PS deprivation with the lowest PS and highest SWS percentages. These data are in line with three earlier studies in rats focused on c-Fos expression in MCH-expressing neurons during sleep. Indeed, MCH-immunoreactive neurons are restricted to the THA and all co-express Nesfatin-1 (MCH-Nesf-1+ neurons) [13], [15]. Two studies observed a very weak activation of MCH+, likely MCH-Nesf-1+ neurons during the recovery period that followed either 3 h of total sleep deprivation [36] or pharmacological treatment with an adenosine A_2A_R agonist [48]. In both cases, PS was almost absent during the sleep recovery (less than 10% of the time spend in PS) essentially composed of SWS. Finally, the third paper reported that around 60% of the MCH+ neurons were Fos-labeled during the profound PS hypersomnia (more than 40% of the time spent in PS) induced by 72 h of PS deprivation [9]. Regarding the flowerpot technique used in this later work, the impact of stress over the course of sleep deprivation on c-Fos expression during PS rebound has often been questioned, as it has also been for other nonspecific patent stressors, including immobilization, daily handling, food and water restriction or physiological discomfort [49]. Moreover, when returned to their home barrel for recovery, rats usually spend about 30–60 min behaviorally awake before the occurrence of the first PS bout. The environmental exploration with sustained motor activity, grooming and food and drink intake that tends to occur during this time are important issues to consider when studying c-Fos expression in hypothalamic networks. To disqualify most of these critical points, we implemented a gentle, short-lasting, less stressful and selective PS deprivation method [50]. Likely supporting this assessment, c-Fos immunostaining was never seen in Nesf-1+ neurons from hypothalamic areas engaged in control of stress or food intake, during either the deprivation or the recovery period. Moreover, we took advantage of the inter-individual heterogeneity of rat’s behavior during recovery to differentiate good-sleepers with a pronounced PS rebound (GS with 31% of PS during the last 2 h) from bad-sleepers showing a weak homeostatic response to deprivation (BS with 14% of PS). Since animals from both groups underwent exactly the same experimental protocol until sacrifice, it is obvious that the activation of Nesf-1+ neurons observed in GS compared to BS rats is independent of the deprivation protocol *itself* and is thus only driven by the homeostatic response induced for PS debt refund during rebound. In conclusion, the present study strengthens our previous work and puts forward additional evidence that the MCH-Nesf-1+ neurons restricted to THA are specifically activated during PS and that their recruitment is correlated to PS amounts. Finally, these functional data fit well with our pharmacological results, which taken together support a functional role for these hypothalamic neurons in PS regulation.

The THA is a very interconnected hypothalamic area suggesting that MCH-Nesf-1+ neurons may possess a large array of post-synaptic receptors [51]. Most of the neurotransmitters tested to date were shown to hyperpolarize MCH+ neurons recorded *in vitro* and spontaneous excitatory post-synaptic currents were relatively scarce compared with inhibitory, likely GABAergic postsynaptic currents [12], [52]. Hence, non-synaptic mechanisms may be evoked to drive these neurons to an active state during PS. These may include hormones, sleep factors or metabolic signals whose local secretion could be positively modulated during PS or when PS homeostasis is challenged. Among them, glucose was a recent focus of interest since it directly depolarizes and increases the excitability of MCH+ neurons through activation of K_ATP_ channels, while it inhibits the co-distributed orexin-secreting neurons within the THA [53], [54], [55], [56]. This suggests an excitatory action of glucose on MCH-Nesf-1+ neurons when body and brain energy resources are high. After feeding, blood glucose is high and arousal is indeed reduced [57]. This is also consistent with the anorectic properties of Nesfatin-1. However, whether glucose concentration is raised during PS in the close vicinity of MCH-Nesf-1+ neurons remains to be assessed.

The identification of downstream targets of MCH-Nesf-1+ neurons that mediate PS-promoting action is a task complicated by our poor current understanding of the signaling mechanisms associated with Nesfatin-1 action. The receptor through which it exerts cellular actions is yet to be identified. Moreover, Nesfatin-1 immunostaining is confined to the cell body cytoplasm and is seemingly absent from axons and synaptic terminals, suggesting post-translational processing of the molecule [15], [58]. It remains that Nesfatin-1 is a secreted molecule in the brain and cerebrospinal fluid [18], [21]. Hence, Nesfatin-1 secreted by THA neurons during PS may reach downstream targets involved in the regulation of vigilance states through either ventricular route, local hypothalamic diffusion with paracrine action or even synaptic release at axon terminals, although evidence of any of these proposed mechanisms is still lacking. Interestingly, whole-cell current-clamp recordings from paraventricular or arcuate nucleus neurons showed Nesfatin-1 to have primarily hyperpolarizing effects [59], [60]. In addition, a large majority of the MCH-Nesf-1+ neurons express GAD, the synthesizing enzyme for GABA [37]. Therefore, Nesfatin-1 neurons within THA are in position to convey inhibitory messengers to downstream targets.

Juxtacellular recordings in rats demonstrated that neurochemically identified MCH+ neurons start firing only when PS is well engaged, thus eliminating their major contribution to the generation of PS *itself*, and the alternation from SWS. Conversely, MCH+ neurons become silent as soon as PS ends [10], when the wake-promoting neurons switch to an active state, as reported for the orexin-containing neurons close to MCH-Nesf-1+ neurons within the THA [61], [62], the histamine-secreting neurons from the hypothalamic tuberomammillary nucleus [63] and the noradrenergic neurons within the brainstem locus coeruleus [64], [65]. This reciprocal firing pattern suggests that the primary role for MCH-Nesf-1+ neurons may be to dampen the wake-promoting systems by reinforcing their inhibition during PS. This mechanism is supported by recent electrophysiological data from anaesthetized rats showing that exogenous Nesfatin-1 strongly reduced the spontaneous firing in more than 70% of the glucose-inhibited neurons recorded within the THA [66]. It is likely that a large portion, if not all of these glucose-inhibited neurons correspond to the THA orexin-containing cells, which promote wakefulness, locomotor activity and foraging [53], [54], [56], [67], [68]. The sustained activation of MCH-Nesf-1+ neurons during PS, especially when homeostatic need is high, would thus result in a consolidation and maintenance of PS by preventing inappropriate re-intrusion of waking. These proposed mechanisms fit with the observations made during PS recovery following selective deprivation characterized by the recruitment/activation of MCH-Nesf-1+ neurons associated with an elevated tone of Nesfatin-1 signaling, an extension of PS bout duration, an increase in PS amounts and a decrease of waking as physiological consequences.

The consistent data from the present study thus support a contribution for Nesfatin-1 signaling in the homeostatic regulation of PS. We thus propose that the Nesf-1+ neurons within THA form an appropriate lever by which the hypothalamus integrates incoming signals to rapidly and precisely adapt the ultradian rhythm of PS (i.e. duration of PS bouts) to physiological or homeostatic needs (e.g. after PS deprivation, learning tasks, food intake and energy expenditure, etc). This action could be done through the inhibition of downstream targets comprising primarily the local hypothalamic wake-active orexin- and histamine-containing neurons.
